# Determination of controlled limit value of groundwater level depth and management practice in Xi’an, China

**DOI:** 10.1038/s41598-020-72523-4

**Published:** 2020-09-23

**Authors:** En-lin Mu, Long Yan, Ai-zhong Ding, Wei Deng, Yong Wang

**Affiliations:** 1 College of Water Sciences, Beijing Normal University, Beijing, China; 2 Water Resources Management Center of Ministry of Water Resources, Beijing, China; 3 China Institute of Water Resources and Hydropower Research, Beijing, China; 4 Engineering Research Center of Groundwater Pollution Control and Remediation Ministry of Education, Beijing, China

**Keywords:** Environmental sciences, Hydrology

## Abstract

Based on the different types of geomorphic units in Xi'an, China, and the groundwater recharge methods of the different geomorphic units, the goal is to ensure the sustainable development and utilization of groundwater, to ensure the natural attributes and to prevent salinization. According to different rainfall conditions, the upper and lower limits of the controlled limit value of groundwater level in different regions are calculated to define the control targets of the different geomorphic units. Based on the calculated controlled limit value of groundwater level and the administrative divisions of Xi'an City, the red lines of groundwater control in each county and city are developed. Four management lines are delineated from the surface to the bottom (from top to bottom, the upper limit of groundwater depth, the upper limit of infiltration of groundwater depth, the lower limit of groundwater depth and the risk line in extremely dry years), and five management areas are delineated (from top to bottom, the prevent soli salinization area, the normal extraction area, the careful extraction area, the permit only in extreme dry years area and the prohibited extraction area) to provide technical support for groundwater management in Xi'an.

## Introduction

Groundwater resources are not only irreplaceable strategic resources for maintaining socioeconomic development but also important factors for stabilizing the ecological environmental system^1^. Due to the unreasonable exploitation of groundwater and the increasingly serious environmental geological problems caused by it, groundwater overexploitation is widespread in the United States, Spain, India, North Africa and the Middle East and other countries and regions. In order to effectively manage the groundwater resources, strengthen the management of groundwater in the overexploited area, and prevent the overexploitation of groundwater in the area without overexploitation, some countries or regions have designated the area according to the local hydrogeological conditions, the current situation of groundwater development and utilization and overexploitation groundwater management zones, such as Arizona groundwater management zone, Kansas key groundwater exploitation control zone, etc.

The Central Shaanxi Plain, where Xi'an is located, is an important grain-producing area in China. Long-term overexploitation and irrational use have led to the serious overexploitation of groundwater, the continuous expansion of the groundwater depression cone, and a series of problems, such as ecological degradation, land subsidence, ground fissures and groundwater pollution, which have become important bottlenecks restricting the sustainable development of the economy and society in the area. The groundwater level and its fluctuation are not only important indexes that reflect the exploitation and replenishment balance of groundwater but also the basis for measuring the rationality of groundwater resource development^2^.To meet the water demands for socioeconomic development and ecological environmental protection, China has been studying the critical groundwater level (buried depth) since the 1960s and using it as a control index to guide, supervise and manage the development and utilization of groundwater resources. The goal of groundwater level management has gradually shifted from the balance of the exploitation and replenishment of the groundwater system to the coordinated and sustainable development of social, economic, ecological and environmental multiuser and multisystem management^3–13^. To protect groundwater resources, Shaanxi Province formulated and implemented the Implementation Plan for Assessment of controlled limit value of groundwater level in Key Areas of Shaanxi Province in 2014. Under the qualification condition that the average decline in a single-well water level is no more than 0.5 m, the analysis and evaluation, assessment of achievement, evaluation of assessment results and formulation of opinions for groundwater monitoring data of various cities (districts) including Xi'an City were performed. However, the evaluation index confirmation lacks a scientific basis, and the groundwater characteristics are not well considered; therefore, it is difficult to interpret the geomorphic type, the relationship between groundwater replenishment and discharge, and the impact of precipitation and human activities on groundwater. Moreover, the current assessment method focuses on the assessment of regional groundwater levels where groundwater levels drop and groundwater overexploitation may occur, but the problems of soil salinization caused by shallow groundwater depth have not yet been considered. These two problems coexist and have seriously affected regional production and development. There is mounting concern about how to support decision makers in driving sustainable groundwater resource management; science needs to support the decision-making process to promote evidence-based decisions^14^. Therefore, the scientific and reasonable determination of groundwater level control indexes is of great significance to improve the level of groundwater management and promote the sustainable use of groundwater in Xi'an.

## Materials and methods

### Study areas

**Figure 1:**
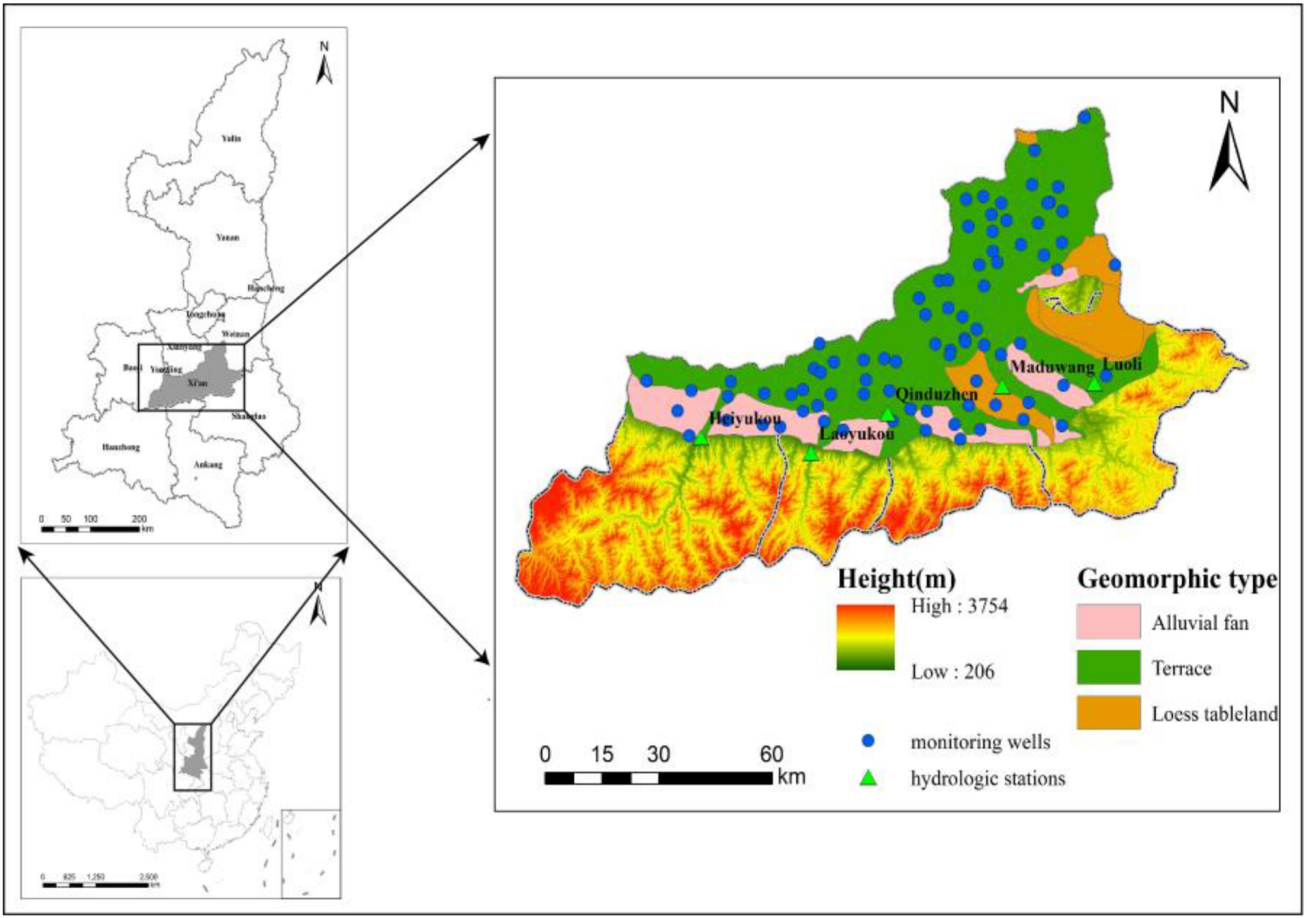
Study area The map were created using ArcGIS 10.2 (https://www.esri.com/sofware/arcgis/arcgis-for-desktop).

Xi'an is located in the Central Shaanxi Basin in the middle of the Weihe River Basin between 107.40°–109.49° east longitude and 33.42°–34.45° north latitude, bordering the Weihe River and Loess Plateau in the north and the Qinling Mountains in the south. Xi'an is approximately 204 km long from east to west and 116 km wide from south to north, covering an area of 9,983 km^2^. According to the Plan for the Delimitation and Protection of Groundwater Over-exploitation Areas in Shaanxi Province, there are 5 overexploitation areas in Xi'an, with an overexploitation area of 601.3 km^2^. Based on the data of the existing monitoring wells, 68 monitoring wells with continuous data are selected as investigation wells for analysis and calculation, including 50 investigation wells in the river terrace, 14 investigation wells in the alluvial fan and 4 investigation wells in the loess tableland (Fig. 1).

The aquifer of terrace, which is mainly distributed on both sides of Weihe River and its tributaries, is composed of sand and gravel rock with a thickness of 10–70 m and a groundwater depth of 3–70 m. The aquifer of diluvial fan, which is mainly distributed in the diluvial sector in the north of Qinling mountains, is composed of sand and sand gravel with a thickness of 10–50 m and a groundwater depth of 5–25 m. The loess tableland is mainly distributed in the Loess Plateau and river valley Alluvium on the northern edge of the Weihe Fault Basin, belonging to the pore and fissure water of the Loess Plateau. The lithology of the groundwater aquifer is mainly loess like sub clay, with a thickness of 50–100 m, and the depth of groundwater level is generally 20–60 m.

### Methods

Based on the different types of geomorphic units in Xi'an and the groundwater recharge methods of the different geomorphic units, the goal is to ensure the sustainable development and utilization of groundwater, to ensure the natural attributes and to prevent salinization. In the alluvial fan and loess tableland area, the continuous flow of rivers from mountainous areas will be ensured, the minimum ecological flow of each tributary will be determined, the relationship between the ecological base flow and groundwater level will be analyzed, and the lower limit value of groundwater pipe control for the alluvial fan and loess tableland area will be determined in combination with the principle of the river channel and groundwater recharge and discharge. For the terrace, the study mainly considers that rainfall can continuously recharge groundwater without cutting off the connection between the rainfall and groundwater. This study also uses the method of soil stratification to calculate the depth when the rate of rainfall infiltration into the wetting front is zero to determine the lower limit value of the terrace underground water pipe control. In addition, the theoretical calculation formula of the maximum height of capillary water rise is used to determine the control index of groundwater depth that causes soil salinization. According to different rainfall conditions, the upper and lower limits of the groundwater level control indexes in different regions are calculated to define the control targets of different geomorphic units. Finally, reasonable groundwater level control indexes for different regions in Xi'an are delimited by using the geomorphic units in the administrative region.

#### The lower limit values of groundwater level depth

##### Terrace

According to the soil water infiltration theory, the critical depth of groundwater recharge by rainfall infiltration is determined, and the key is to analyze the process of the redistribution of rainfall infiltration and soil water after infiltration. According to the saturated and unsaturated infiltration theory, infiltration is divided into two stages, namely, rainfall infiltration and soil water redistribution, and a model for calculating rainfall infiltration depth is established. In the stage of rainfall infiltration, soil water movement adopts the saturated soil water movement method. When soil water enters the redistribution stage, the infiltration depth in the redistribution stage of soil water is calculated via soil stratification by using the unsaturated soil water movement method.

**Rainfall infiltration stage** Based on the saturated infiltration theory and water balance principle:

$$ H_{P} = \frac{F}{{\theta_{s} - \theta_{1} }}/1000 $$

where *H*_*p*_ is the position of the wetting front during saturated infiltration, m; *F* is the rainfall infiltration, mm; *θ*_*s*_ is the saturated soil water content, cm^3^/cm^3^, expressed as a percentage; and *θ*_*i*_ is the initial soil water content, cm^3^/cm^3^, expressed as a percentage.

**Soil water redistribution stage** Using the unsaturated infiltration theory and on the basis of Darcy's law of unsaturated soil movement, each soil layer is taken as a calculation unit according to the method of soil stratification, the upper part of the soil layer is the wetting front position, and the water content of the soil layer is the initial water content. When the movement of a unit is finished, it enters the lower boundary of the unit, that is, the upper boundary of the next calculation unit. At this time, the wetting front has moved downward by one layer, and the soil water content above the wetting front is still uniformly distributed in an unsaturated condition. Affected by the potential difference in the gradient, the wetting front continues to move until the wetting front enters the next calculation unit at a certain moment with a negative moving speed, i.e., stops moving downward. At this time, the moving depth is the maximum infiltration depth in the redistribution stage.

Based on the conditions of the soil water content, the basic calculation formula is as follows:

$$ \begin{aligned} \nu = & - \frac{{K(\theta_{i - 1} ) + K(\theta_{i} )}}{2}\left[ {\frac{{\psi_{m,i} - \psi_{m,i - 1} + \psi_{g,i} - \psi_{g,i - 1} }}{\Delta z}} \right] = \frac{{K(\theta_{i - 1} ) + K(\theta_{i} )}}{2}\left[ {\frac{{h_{m,i} - h_{m,i - 1} + \Delta z}}{\Delta z}} \right] \\ \theta_{t} = & \theta_{t - 1} \frac{{h_{t - 1} }}{{h_{t - 1} + \Delta z}} \\ \end{aligned} $$

where *v* is Velocity of wetting front; *K* is Unsaturated hydraulic conductivity; *Ψ*_*m*_ is soil matrix potential; *Ψ*_*g*_ is gravitational potential; *θ*_*t*_ is the soil water content at the top of the wetting front at time t; *θ*_*t−1*_ is the soil water content at the top of the wetting front at time t−1; and *h*_*t−1*_ is the thickness of the wetting layer at time t−1; where *h* is calculated by the van Genuchten formula and is shown as follows ^15^:

$$ h = \frac{1}{\alpha }\left[ {\left( {\frac{{\theta_{s} - \theta_{r} }}{{\theta - \theta_{r} }}} \right)^{\frac{1}{m}} - 1} \right]^{\frac{1}{n}} $$

where *h* is the soil suction, cmH_2_O; *θ*_*s*_ is the water content of the saturated soil; *θ*_*r*_ is the water content of the saturated wilting soil; *θ* is the actual soil water content; and $r = [1.581(n - 39.5\% ) + 0.079] \cdot d$, m, n is parameter.

**Alluvial fan** In the dry season, the river has a low and stable minimum runoff period without rainfall. At this time, the river only depends on groundwater recharge. If the groundwater depth is too low, there will be an ecological disaster or even a cutoff. To maintain the ability of the river to survive in the dry season, indexes, such as the multiyear average water level of the river corresponding to the multiyear average flow, the minimum ecological water level of the river corresponding to the minimum ecological flow, and the river bottom elevation, are adopted as the lower limit control indexes of the groundwater.

The river bottom elevation directly refers to the large section data of each river. According to the daily river runoff observation data of the main tributary of the Weihe River, the multiyear average flow and minimum ecological flow of the hydrological station section of each river are determined. Second, the water-level-discharge relation curve of the main tributaries are obtained by fitting the water level and discharge data of each hydrological station, through which the multiyear average water level and the minimum ecological water level of the hydrological station section of each river are determined accordingly. Finally, considering the management needs and the most extreme situation, the multiyear average water level and the minimum ecological water level of the hydrological station section of each river are converted to the edge of the alluvial fan through the regional topography and river gradient of the alluvial fan, and the lower limit control indexes of the groundwater in the relevant counties and cities in the alluvial fan regions are obtained through the superposition of the administrative divisions.

Based on the geomorphic distribution and hydrologic station distribution in Xi'an, 4 main branches of the Weihe River are totally selected in the calculation to be used in the lower limit control index calculation of groundwater in the diluvial fan areas, which are respectively the Heihe River, the Bahe River, the Fenghe River and the Laohe River. Besides, main control hydrologic stations in these rivers are taken as the calculation benchmarks, and the control points are set and converted to the diluvial fan edge as the groundwater control index in related counties and cities of the diluvial fan area.

##### Loess tableland

Based on the geomorphic distribution and hydrologic station distribution in Xi'an, Luolicun and Mawangdu hydrologic station are totally selected in the calculation to be used in the lower limit control index calculation of groundwater in the loess tableland areas.

The method for the calculation of the loess tableland is basically the same as that of the alluvial fan area, and the main calculation parameters and influencing factors are also the same as those of the alluvial fan area. The determination method for the minimum ecological flow and the average flow of many years are consistent with that for diluvial fan area, and Luolicun is used as the control hydrologic stations of diluvial fan area. Therefore, the minimum ecological flow of Maduwang Station is calculated below. The final average flow and the minimum ecological flow of each station for many years in the area of loess terrace like plain are shown in Table 1.

**Table1:** The maximum infiltration depths of rainfall under different conditions.

Soil water content	7.5%	15%	25%
**Rainfall (mm)**			
430.78	6.88	4.24	3.7
594.47	9.5	5.87	5.11
724.63	11.58	7.14	6.21

The average water level and the minimum ecological water level of the section in each hydrologic station for many years are uniformly converted to the loess tableland edge by river slope and based on topographic conditions. According to the previously listed administrative region-river correspondence, the final lower limit control index of groundwater in the loess tableland area is shown in Table 2.

**Table2:** Average flow and the minimum ecological flow of hydrologic stations.

Hydrologic station	River	Average flow (m^3^/s)	Minimum ecological flow (m^3^/s)
Heiyukou	Heihe River	16.62	1.58
Luolicun	Bahe River	5.96	0.61
Qinduzhen	Fenghe River	7.49	0.74
Laoyukou	Laohe River	3.52	0.32
Mawangdu	Bahe River	14.05	1.39

#### The upper limit values of groundwater level depth

The upper limit values of groundwater level depth is set to prevent the salinization of the region caused by the high groundwater level. The key to calculating the upper limit control index is to determine the rising height of phreatic water evaporation, that is, the maximum possible rising height of the capillary water.

Based on capillary theory, Laplace proposed the calculation formula of capillary rising height as follows:

$$ \frac{h}{r} = 2\left( \frac{a}{r} \right)^{2} \cos \theta $$

where *h* is the rising height of the capillary water; *α*is a parameter; *γ* is the pipe diameter; and *θ* is the contact angle between the liquid and pipe wall.

For soil water, the surface tension is related to air temperature, the soil properties are available in a prepared table and the density and gravitational acceleration of water are known. When the capillary rises to its highest point, the surface of the capillary water contacting air is spherical under the action of surface tension, so the water–air contact angle is 0°. The pore diameter of the soil capillary is the equivalent pore diameter R of the soil, and the formula for calculating the maximum capillary rising height H can be expressed as follows:

$$ H = \frac{2\gamma }{{\rho gR}} $$

The key parameter to be determined in the theoretical calculation formula of the maximum lifting height of capillary water is the effective pore diameter of soil (namely equivalent pore diameter).

The formula for calculating the relationship between effective pore diameter of soil and soil particle size;

$$ r = [1.581(n - 39.5\% ) + 0.079] \cdot d $$

where *r* is effective pore diameter; n is soil porosity; d is oil particle size.

Through above formula, the reasonable soil structure can be selected based on the known soil porosity and under the condition of effective particle size to calculate the effective pore diameter, so as to provide reliable parameters for the theoretical formula of capillary to calculate the lifting height of capillary water.

#### Risk limit values of groundwater level depth

Considering the influence of the different combinations of rainfall and soil moisture on rainfall infiltration and salinization prevention, three management lines are delineated from the surface, namely, the upper limit control value of groundwater depth (critical upper limit control value of groundwater depth without salinization), the early warning value of groundwater depth and the lower limit control value of groundwater depth (maximum rainfall infiltration depth under normal and extreme conditions). In light of the special drought years, a risk defense line is established.

When the groundwater depth exceeds the control value, excessive exploitation and imbalance will occur, and the river will shrink. Groundwater will be under the control value for a long time, and even the flow will be interrupted. In extremely dry years, the groundwater level is allowed to break through the lower limit control value and set the risk defense line (1–2 m for the loess tableland and 3–5 m for the alluvial fan) to allow declines within the risk line, but the groundwater level cannot continue to cross the boundary. When the water supply condition improves, the groundwater level should rise above the control line. The development and utilization of groundwater should be controlled as much as possible in this area, pressure extraction measures should be taken into account to control the downward trend of the groundwater depth, and the downward range should be reduced annually until the groundwater level is increased to the lower limit.

## Results and discussion

### The lower limit values of groundwater level depth

#### Terraces

1. Rainfall
  The prefectural and municipal rainfall is obtained by spatial interpolation, and provided with frequency arrangement analysis. 10%, 50% and 95% of rainfall in main rainfall months (April–October) respectively in high flow year, median water year and dry year are selected as the infiltration capacity, which are respectively taken as 724.63 mm, 594.47 mm and 430.78 mm.
2. Soil water content
  The cinnamon soil mainly exists in the research area. The saturated water content of soil is related to the soil porosity. Through field investigation and look-up in the Chronicles of Soil Species in Shaanxi Province, the porosity of cinnamon soil is obtained. The saturated water content of soil is determined based on the porosity of cinnamon soil, which is taken as 47.2%.
  The concepts of drought in agriculture are introduced in this research, and the extra dry water-holding capacity, water capacity without drought and field capacity in the Grade of Agricultural Drought are taken, and respectively used as dry, general and humid soil moisture conditions. According to the definitions in the Grade of Agricultural Drought, the relative humidity of extra dry water-holding capacity, water capacity without drought and field capacity is respectively 30%, 60% and 100%. According to the Chronicles of Soil Species in Shaanxi Province, the field capacity of cinnamon soil is 25%. Finally, the initial water contents of cinnamon soil in three soil moisture conditions are respectively 7.5%, 15% and 25%.
3. Hydraulic characteristic parameters of soil

During the calculation by the Van Genuchten formula, some hydraulic characteristic parameters of soil shall be determined as well. The empirical value of cinnamon soil given by Zhang Weizhen^16^is referred to in this research. *Ks*, *a*, m and n are respectively taken as 0.26 m/day, 0.01, 0.5 and 2.

Considering the fundamental assumption of soil layer-by-layer calculation in the redistribution process, with thinner soil layer, the fundamental assumption of uniform motion in each layer is more close to the actual situation. In this research, the soil layer thickness is taken as 0.01 m.

The rainfall infiltration depth calculation model is applied to respectively obtain the maximum infiltration depths of rainfall under different incoming water conditions and different soil moisture conditions, as shown in the Table1.

#### Loess tableland and alluvial fan

In this research, the day-by-day river flow materials (1980–2015) measured at each computational river control hydrologic station are selected for analysis, to obtain the minimum ecological flow after the calculation of each hydrologic station section. The average flow of many years has reflected the flow state of rivers under general conditions. The day-by-day river flow materials (1980–2015) measured at each computational river control hydrologic station are selected for analysis as well. The final average flow and the minimum ecological flow for many years of each station are shown in Table 2.

**Figure 2:**
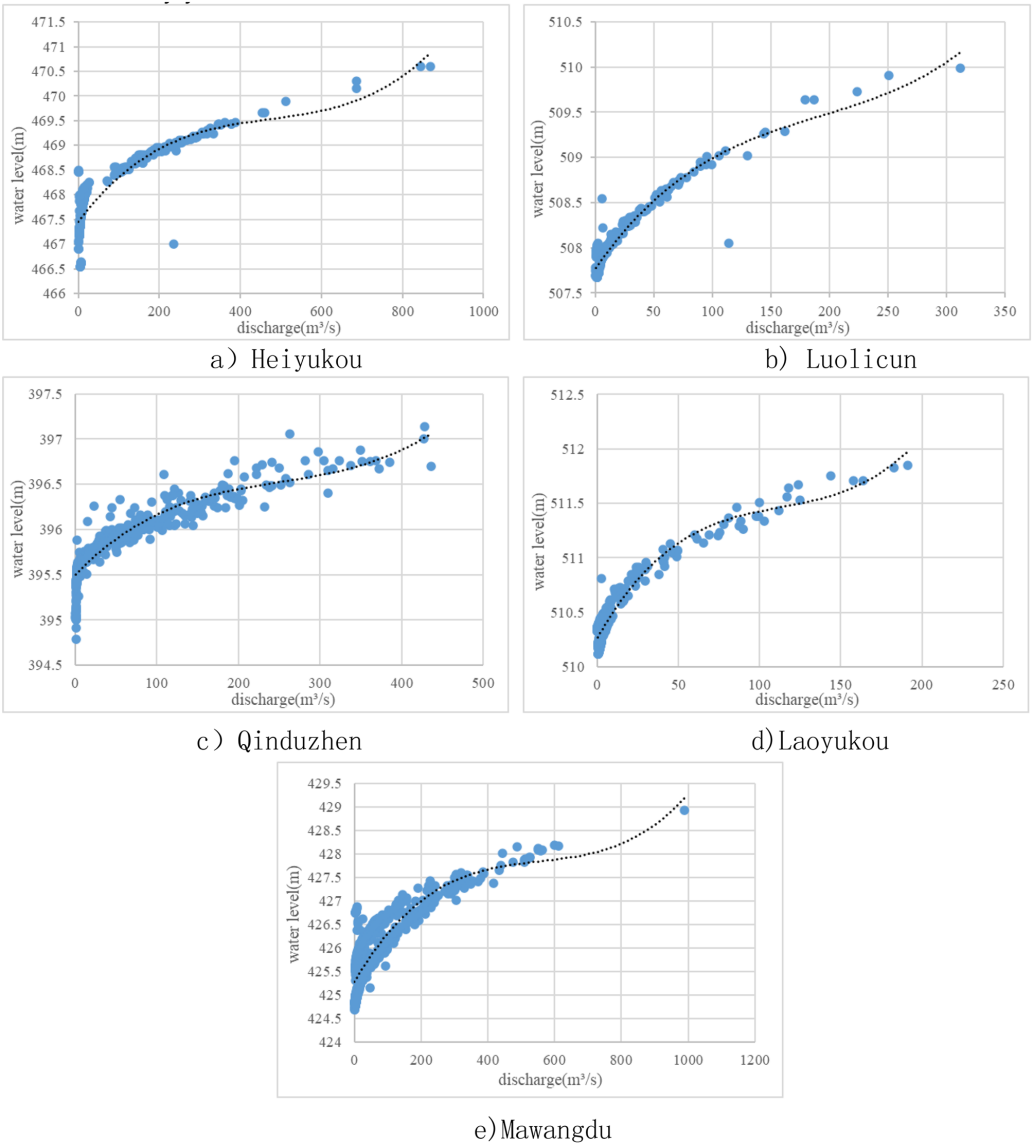
Relation curve of stage-discharge in each station.

For stable channels, there is a certain correspondence between water level and flow. The relation curve of stage-discharge in each station is obtained by three spline curve fitting through the measured flow and water level data (2006–2015) of each computational river main control hydrologic station. The relation curve of stage-discharge in each station is shown in Fig. 2. According to the relation curve of stage-discharge in each station, the average flow and the minimum ecological flow of many years are respectively converted into the average water level and the minimum ecological water level of many years.

In order to calculate the lower limit control index of groundwater, considering the worst situation, the average water level and the minimum ecological water level of the section in each hydrologic station for many years are uniformly converted to the diluvial fan edge by river slope and based on topographic conditions. The final lower limit values of groundwater level depth in the diluvial fan area is shown in Table 3.

**Table 3:** The lower limit values of groundwater level depth.

Region	River bottom elevation (m)	Average water level (m)	Minimum ecological water level (m)
**Diluvial fan area**			
Zhouzhi	401.05	406.08	405.50
Huyi	388.69	389.94	389.67
Changan	407.34	410.25	410.08
Lantian	404.82	406.14	405.84
Shiqu	404.82	406.14	405.84
Changan	415.30	415.58	415.43
Shiqu	377.30	377.58	377.43
Lantian	404.94	406.24	405.84
Lintong	394.94	396.24	395.84
Yanliang	365.95	366.20	366.08

### The upper limit values of groundwater level depth

The cinnamon soil is the main soil in the calculation area. Based on the descriptions for cinnamon soil characters in the Chronicles of Soil Species in Shaanxi Province, and combining the actual investigation results, the volume-weight, porosity and effective particle size of cinnamon soil are respectively determined as 1.49 g/cm^3^, 47.2% and 0.045 mm.

Based on the field visit, the soil temperature of the cinnamon soil area in the Guanzhong Region is about 13.1–18.7 ℃, and the surface tension is taken as 74 (10^−3^ N/m).

Combining the particle sizes of different soils and the relationship between soil particle size and effective pore diameter, the effective pore diameters of three types of soil are calculated, and the lifting heights of capillary water of different soils are calculated based on the theoretical formula of capillary water lifting height, namely the upper limit control index of groundwater, 1.67.

## Conclusions

Based on the analysis of the groundwater recharge modes of the different types of geomorphic units (river terrace, loess tableland and alluvial fan) in Xi'an, the groundwater control indexes in different regions are calculated in combination with different rainfall conditions and soil conditions to ensure the sustainable development and utilization of groundwater, guarantee natural attributes and prevent salinization. As seen from the protection objective, the limit control index of groundwater is determined based on the critical depth of salinity and is controlled based on the maximum rise height of capillary water, which is approximately 1.2–1.6 m. Considering the maintenance of the integrity of the hydrological cycle and the assurance that the connection between the surface water and groundwater is not damaged as the determining basis for the lower limit control index of the groundwater, the inter-river terrace, loess tableland and alluvial fan are considered due to the differences in the replenishment relationship between different hydrogeological types and different geomorphic units. The hydraulic gradient of terrace groundwater is small, and the lateral recharge is slow, mainly to ensure the stability of vertical rainfall on groundwater recharge. The critical depth of groundwater recharge by infiltration, which is 6–8 m under normal conditions and can reach 10–12 m under extremely favorable conditions, is controlled. Lateral recharge from the slope plays a leading role in the alluvial fan, the groundwater hydraulic gradient is large, and the groundwater and surface water are closely connected laterally. As the surface of the loess tableland is covered with a thick loess layer, the vertical recharge by rainfall is small, and the lateral recharge of the river course is the main factor. Both vertical and lateral recharge mainly ensure the horizontal connection of surface water. The main tributaries of the Weihe River are continuously flowing, and the ecological water level of the river (taking the river bottom elevation, average annual discharge and minimum ecological flow into account) is used for control. The specific control of the buried depth is related to the actual topography.

Based on the calculated groundwater control index and the administrative divisions of Xi'an City, the red lines of groundwater control in each county and city are developed. Four management lines are delineated from the surface to the bottom (from top to bottom, the upper limit of groundwater depth, the early warning value of groundwater depth, the lower limit of groundwater depth and the risk line in extremely dry years), and five management areas are delineated (from top to bottom, the saline-alkali area, the normal extraction area, the careful extraction area, the discontinuous overextraction area under special drought conditions and the prohibited extraction area). For the terrace, the four management lines are the critical upper limit control value of groundwater depth without salinization from top to bottom, which is approximately 1.2–1.6 m, the maximum depth of normal rainfall infiltration, which is approximately 6–8 m, the maximum depth of rainfall infiltration under extreme conditions, which is approximately 10–12 m, and the risk line of defense (the maximum depth of rainfall infiltration under extreme conditions, which is approximately 2 m). For the loess tableland and alluvial fan, the four management lines are the upper limit control value of groundwater depth without salinization from top to bottom, which is approximately 1.2–1.6 m, the average annual water level of the course (early warning), the river bottom elevation (management and control), and the risk line of defense (the alluvial fan is approximately 3–5 m from the bottom of the river and approximately 2 m from the loess tableland).
